# Could an increase in vigilance for spinal tuberculosis at primary health care level, enable earlier diagnosis at district level in a tuberculosis endemic country?

**DOI:** 10.4102/phcfm.v10i1.1666

**Published:** 2018-06-18

**Authors:** Karen M. Galloway, Romy Parker

**Affiliations:** 1 Private Practice, Centani, South Africa; 2 Department of Anaesthesia and Perioperative Medicine, University of Cape Town, South Africa

## Abstract

**Background:**

Expert clinicians and researchers in the field of spinal tuberculosis (STB) advocate for early identification and diagnosis as a key to reducing disability, severity of disease, expensive surgery and death, especially in tuberculosis (TB) endemic countries like South Africa. South Africa has the highest incidence per capita of tuberculosis in the world, and a conservative estimate of the incidence of STB in South Africa is 8–16:100 000. People living with STB may initially present to primary health care (PHC) centres, where the opportunity exists for early identification. Spinal pain is the most common presentation of STB, but even this symptom may not be present. Occasionally the only symptoms are neurological injury, dysphagia or referred pain. Computerised tomography-guided biopsy remains the diagnostic gold standard for STB.

**Aim:**

A narrative review was undertaken to investigate the evidence available that could assist with the early diagnosis of STB.

**Method:**

Articles were searched for and retrieved from three databases and assessed for quality and relevance to primary settings in a TB endemic country.

**Results:**

The following evidence-based, affordable and available tools could facilitate early diagnosis of STB at PHC and district hospital levels: (1) back pain screening questions, undressed spinal physical examination, HIV and antiretroviral therapy history, (2) erythrocyte sedimentation rate, C-reactive protein, platelets, haemoglobin, white cell count (WCC), sputum for GeneXpert and accurate weight measurement, (3) physiotherapy and/or medical and/or speech therapy assessment, (4) full spinal radiograph, chest radiograph, abdominal ultrasound, urine lipoarabinomannan (LAM) if CD4 < 200 and ultrasound-guided biopsy of superficial abscesses, (5) clear referral guidelines at all levels, (6) a positive response to treatment to confirm the diagnosis.

**Conclusion:**

These affordable and simple actions at PHC and district levels could facilitate earlier diagnosis of STB.

## Introduction

Despite expert agreement about the importance of early identification of spinal tuberculosis (STB), strategies to increase the clinical suspicion of the condition are lacking, and there is a paucity of literature informing the primary health care (PHC) practitioner of diagnostic strategies to use in resource-poor settings. The presented concept arose from 80 years of clinical experience in a deeply rural setting in South Africa with limited access to computerised tomography (CT) scan, magnetic resonance imaging (MRI) scan or CT-guided biopsy.

The World Health Organization (WHO) *Global Tuberculosis Report* of 2016 listed South Africa as having the highest incidence of tuberculosis (TB) per capita in the world, with an estimated incidence of 834 per 100 000.^1^ Based on the prevalence of STB in Danish (1.9%) and Taiwanese (1.2%) studies, it can be estimated that the incidence of spinal tuberculosis (STB) in South Africa is 8–16 per 100 000.^2,3,4^ The actual incidence of STB may be higher, as the introduction of improved diagnostic techniques such as full-spine MRI and nucleic acid amplification techniques (GeneXpert) has resulted in more diagnoses, as well as identifying more atypical spinal tuberculosis (STB), which was previously difficult to see with plain radiography.^5,6^ Furthermore, the influence of human immunodeficiency virus (HIV) on STB most likely contributes to a higher incidence than that suggested.

The pathogenesis of HIV and TB are intimately linked, as HIV impedes a person’s ability to mount an immune response to TB infection, and a TB infection stimulates an immune response, resulting in increased replication of HIV.^4^ It is suggested that 1% – 2% of people with TB, without HIV co-infection, develop STB, compared to 30% of people with HIV co-infection.^4,7,8^ HIV also influences the presentation of STB, with one study showing a trend towards a greater epidural abscess volume but significantly lower incidence of vertebral collapse in people with concurrent HIV and STB infection.^4^ These findings suggest that people living with HIV and STB may have less evidence on plain radiography despite more vertebral sites being affected.^9^ Antiretroviral therapy (ART) decreases TB risk by 65%.^10^ However, immune reconstitution syndrome (IRIS) also plays a role in STB, as those who are unknowingly co-infected and initiating ART, are at risk of developing active symptoms of TB. This is particularly the case for those who may have STB with no constitutional symptoms nor symptoms of classic pulmonary tuberculosis prior to ART initiation.^11^

In order to define an early diagnosis of STB, it is helpful to have a method for classification. Tuli’s classification is applicable to the PHC setting.^12^ It defines four grades with the earliest grade, grade I (negligible neurological impairment), presenting with signs of upper motor neuron damage whilst the patient is unaware of neurological deficit.^12^ The American Spinal Injury Association (ASIA) scale can be used for more details on neurological involvement. There is not one classification that covers all types of STB, creating difficulty in having a single standard to define early.

### Cost-effectiveness of early diagnosis and public health benefit

The early identification of STB has cost benefits to patient and state, as a delay in diagnosis of STB is directly linked to the degree of disease severity, disability and death.^13^ Cost benefits of early identification include decreasing the severity of disability and length of hospital stays, as well as preventing surgery, because early identification allows for successful medical treatment in most cases.^14,15,16,17^ Pulmonary TB (PTB) (not necessarily symptomatic or diagnosed) is present in 15% – 31% of people with STB.^2,18,19^ Furthermore, the incidence of Multi-Drug Resistant Spinal Tuberculosis (MDR STB) in South Africa is currently reported to be 5.8% but has been reported to be as high as 11.8% in other settings.^6,10^ Thus, early identification of STB has the potential to contribute to early appropriate treatment for both drug-sensitive and resistant TB and to prevent further spread via a pulmonary focus.^13,20^

### Gold standard diagnostic methods: Computerised tomography-guided biopsy

The gold standard method for the diagnosis of spinal *Mycobacterium tuberculosis* (*Mtb*) is the identification of the mycobacterium through culture or classic histological findings after CT-guided percutaneous biopsy or surgical biopsy.^21^ CT-guided biopsy allows isolation of the pathogen from a selection of possible causative organisms, as well as testing for drug sensitivities.

MRI, although not the gold standard, is a secondary, sensitive imaging technique that can reveal the extent of the disease earlier and more accurately than radiographs and with better visualisation of soft tissue than a CT scan.^4,5,22^ MRI evidence of STB can support suspicions of STB in the absence of CT-guided biopsy or in the case of difficulty isolating an organism in a biopsy; however MRI alone is not able to differentiate between cancers or other pathogens.^23,24^ MRI can be used to identify suspected STB lesions in people with PTB and thus ensure that they are not inadequately treated with only 6 months of treatment.^22^ However, even in these cases biopsy is recommended, as a person with PTB could have a spinal lesion that is not caused by *Mtb*.^25,26^

In resource-poor contexts, South Africa included, access to diagnostic MRI is poor and access to diagnostic CT-guided biopsy even more difficult.^21,24,25,27^ Despite the scarcity of gold standard diagnostic techniques, there are clinical tools that are available to assist the primary care clinician in their clinical reasoning.^21,28^

## Methods

**FIGURE 1:**
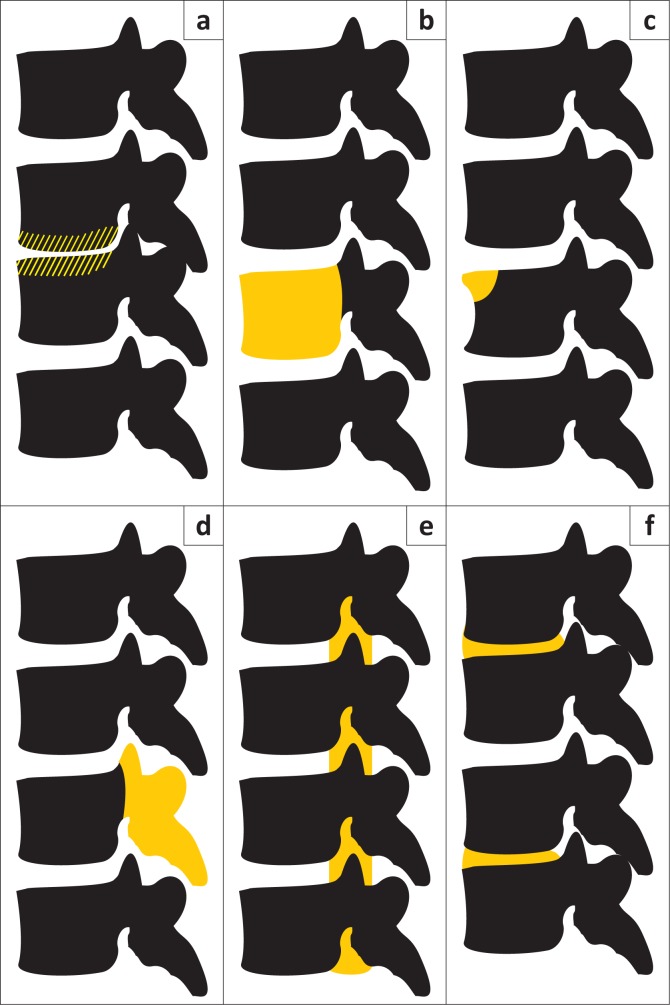
Locations of spinal tuberculosis: (a) paradiscal (typical); (b) central; (c) anterior; (d) posterior; (e) extradural tuberculoma; (f) skip lesions.

The aim of this narrative review was to explore the research around early identification of STB at the PHC and district levels, with the purpose of making some recommendations for practice towards earlier diagnosis in order to prevent disability and death. The specific objective was to identify diagnostic methods appropriate for use at the PHC and district levels to enhance the early diagnosis of STB to reduce disability and death. A literature search of PubMed, Medline and Google Scholar was conducted for articles published in English from 1997 to 2017 using the following search terms: spinal tuberculosis and/or tuberculous vertebral osteomyelitis and/or TB spine and/or tubercular spondylitis and/or tuberculosis of the spine and/or skeletal tuberculosis and/or Pott’s disease and/or tuberculous spondylodiscitis’, ‘early’ and diagnosis and/or screening and/or identification and/or detection. Articles were eligible for inclusion if they were original studies that reported on more than five cases and used gold standard diagnostic methods. Additional articles were included if they reported on assessment and management of STB in a PHC or district hospital setting, were from countries recognised as having a high burden of disease or dealt specifically with early identification or diagnosis. In addition, a hand search was conducted to identify relevant documents including TB guidelines and policies internationally and specific to South Africa. Hand searching of reference lists of identified articles led to identification of further articles. In total 49 original research articles and 33 review, opinion or case study articles were considered relevant for this review (Figure 1). Following screening of the articles to ensure they met the inclusion criteria, the primary author extracted data into a spreadsheet categorising studies as per Table 1 and summarising findings. As this was a narrative literature review, ethical clearance was not required.

**TABLE 1:** Description of articles included in review.

Articles included in review	Number of articles
Total articles included in review	82
Specifically addressing ‘early’ diagnosis	4
Research located in high burden countries	45
Gold standard diagnostics used – positive culture from CT-guided biopsy	29
Research located in PHC or district hospital setting	2
Original research: Retrospective quantitative study	41
Original research: Prospective quantitative study	8
*N* = 0–10	12
*N* = 11–50	20
*N* = 51–100	7
*N* = 101–1000	17
*N* = > 1000	2
Original research: Qualitative	0
Case studies	11
Review articles	15
Opinion pieces	7

PHC, primary health care; CT, computerised tomography; *N*, case numbers in each study.

## Results

Most STB research conducted has used retrospective record review methods, with eight articles reporting on prospective case series. Typically, the sample sizes were small (<300). Forty-five per cent of studies used organism-proven gold standard diagnosis; however, researchers in low-resource settings relied on suggestive imaging reports or response to treatment to support the diagnosis of STB. Despite the general absence of gold standard diagnostics in low resource settings, studies are informative regarding what is possible in TB-endemic areas with resource limitations, pending access to the requisite gold standard. All articles used were quantitative research and qualitative data, and patient perspectives on this topic were dramatically absent. The clinical methods reported on in this article to facilitate the early diagnosis of STB are supported by culture-proven studies unless otherwise stated.

### Accessible tools that can assist in raising suspicion about spinal tuberculosis

It is easier to make a diagnosis of late classic paravertebral type STB with wedge compression fractures than of early STB. However, this article proposes that early STB could be identified more often if the health worker considered all the possible information as part of the clinical reasoning process. In this article, we will consider the tools available to clinicians at different levels of care (primary, PHC outreach and support, district hospital levels) and discuss the literature supporting their use. There is no single test for STB (even CT-guided biopsy may yield no pathogens because of STB being a paucibacillary disease), and all of the following diagnostic tests serve only to contribute to the clinical reasoning of the clinician and occasionally to assist in ruling out other differentials.

### Tools at primary care

#### Understanding why the patient is presenting

Back pain is a very common presenting complaint at primary care level, and it is vital to separate pathological back pain from regular mechanical back pain, whilst managing busy clinics.^29^ The *Adult Primary Care Guidelines* of 2016–2017 recommend standard subjective and objective techniques for use in the initial assessment of a patient,^30^ including important information about the patient’s beliefs, fears and concerns. We propose two initial questions that may add information: ‘Please tell me more about your back pain and why you are here today?’ and ‘Do you have specific reasons to be more concerned about this back pain than you would about other kinds of back pain?’ These could highlight elements of the objective assessment that need closer scrutiny.

When the patient is given time to answer these suggested questions, the clinician is then able to listen for symptoms that may indicate STB. Symptoms of STB are notoriously unspecific and, although certain symptoms can be present, their absence does not exclude the diagnosis. The most common presentation of STB is spinal or referred radicular pain in 85% – 100% of cases,^5,17,18,29,31^ with the highest proportion of cases presenting in the lumbar, thoracic or thoracolumbar regions, followed generally by cervical, lumbosacral, cervicothoracic, sacral or craniocervical.^3,31,32,33^ In 24% – 56% of cases, constitutional symptoms are present,^5,17,19,21,29^ with weight loss reported in 36% – 48% of cases.^5,32^ Neurological signs (indicative of more progressive disease) are reported in 21% – 85% of cases.^5,18,21,31,32,34^

One study from China including 89 people with mild STB in a cohort of 740 with STB provides further information on symptoms that may assist in the identification of STB. The criteria for the classification early in this study included, in some ways, quite advanced disease, with amongst others mild neurology, a kyphosis or gibbus of ≤ 30° and up to three levels of vertebral involvement. The presenting symptoms of these early cases were spinal pain at the site of infection (97%), night sweats (37%), low grade fever (28%), weight loss (44%), restricted movement (90%), percussion pain (99%), local deformity (11%) and sinus (2%). This study accepted cases that were diagnosed on response to treatment without identification of *Mtb*.^15^ The well-informed clinician will recognise that many of the above symptoms are also associated with other pathologies.

#### Differentiating pathological back pain

Red flag questions used in isolation, in the absence of clinical reasoning around the full context of a patient, have been questioned, even in regard to screening for spinal malignancy.^35^ The risk factors that need to be in the forefront of each health care worker’s mind in a TB endemic country are as follows: children complaining of back pain, immunocompromising states (pregnancy, being elderly, drug and alcohol abuse, steroid treatment, post–organ transplant and poverty), immunocompromising illnesses (HIV, diabetes mellitus, renal failure, rheumatoid arthritis, cirrhosis, Systemic Lupus Erythematosus (SLE) and cancer), back pain > 3 months, age < 20 or > 50, bladder or bowel dysfunction, saddle paraesthesia and a history of cancer.^5,19,20,36,37,38,39,40^ Details about the start date of ART and of the level of immune system compromise at that date enables evaluation of the risk of an unmasking IRIS, which can present within months after starting or changing ART.^11,32^

In the diagnostic process, the clinician must consider the following conditions: pyogenic or fungal vertebral osteomyelitis, brucellar spondylitis, multiple myeloma, lymphoma, metastatic disease, osteoporotic vertebral fractures, atypical degenerative disc disease, sarcoidosis, other primary tumours (acute myeloid leukaemia, angiosarcoma, angioblastoma, giant cell tumour, osteochondroma) or aneurysmal bone cyst.^24,27,41,42^

#### The physical examination: Having a careful look

Observing the site of pain and ability to move at that area (with the patient undressed) is accepted as part of a minimal assessment and is also particularly pertinent to the diagnosis of STB.^13^ We suggest that localised point pain indicative of a STB lesion is often identified by the patient demonstrating the site of pain with a single finger as opposed to a whole hand. Zhang reported percussion pain as one of their positive findings in people with early STB.^15^ Basic observation includes an accurate assessment of the patient’s weight to identify weight loss. Weight gain can be used later as an indicator of response to treatment.

Following the completion of the standard subjective and objective examination of the patient, several additional tests are recommended in the literature for use in patients where there is a concern of STB; these include sputum and HIV testing, Erythrocyte Sedimentation Rate (ESR), C-reactive protein (CRP), platelets, white cell count (WCC) and alkaline phosphatase.^29^ These are useful in the diagnosis of STB, although they are not diagnostic of STB in themselves. The high clinical utility of blood tests means these can be applied at primary clinic level with results reviewed on a return visit, thus reducing diagnostic delays and improving affordability and patient accessibility.

#### Special tests: If there is concern about spinal tuberculosis

**Sputum:** As 14% – 37% of people with STB are co-infected with PTB,^9,19,29^ sputum or an induced sputum (using a nebuliser in the open air with 3% hypertonic saline if negative pressure rooms are not available) should be sent for culture and analysed with GeneXpert.^43^ A positive sputum indicates the need for TB treatment, but further exploration is necessary to determine the length of treatment required. It must be noted that a negative sputum result does not rule out STB.^2,3,17,44,45^ Therefore, a sputum sample should be obtained from every patient in whom STB is suspected.

**HIV, CD4, antiretroviral therapy history and viral load:** Because of the increased risk that concomitant HIV poses, an HIV test is indicated for patients at risk or in those who do not know their status. A positive status indicates the need to assess their CD4 levels. If the patient is taking ART, it is important to assess viral load for viral suppression. If the patient has recently started ART or changed regime, he or she has an increased risk of IRIS.

**Erythrocyte sedimentation rate, C-reactive protein and platelet count:** A raised ESR can assist in diagnosis and can be used to monitor response to treatment in culture-proven cases, but it is not specific to STB.^5,14,19,24,31,32,34^ A point-of-care ESR is possible at certain PHC clinics with test results available within one hour, removing the need for a repeat visit to the health facility. However, the interpretation of an ESR needs to be carefully considered for several reasons. The accuracy of an ESR might be affected by room temperature^46^ and HIV affects ESR, possibly obscuring the results.^26,33,47^ However, Daniel and Dunn reported no difference in the ESR of 57 HIV-infected STB patients compared with 21 STB patients uninfected with HIV. Therefore ESR is still recommended as a useful diagnostic clue and indicator of response to treatment, even in HIV positive patients.^48^

Finally, it must be noted that an ESR may be increased for reasons other than STB, and a low ESR does not exclude STB (normal ESR reported in 11% – 12% of cases).^5,19^ As an alternative inflammatory marker, CRP may also be useful to inform diagnosis. A raised CRP has been observed in between 90% and 100% of STB cases but, as with the ESR, it is not specific to STB and may indicate some other inflammatory or infective process.^9,19,29,48^ In addition, a raised platelet count has been observed in people with STB, in both those infected and uninfected with HIV.^48^ Therefore ESR, CRP and platelet count are useful tests to assist in the clinical reasoning around a patient presenting with spinal pain, suggesting that an infective process is present.

**White cell count, haemoglobin and alkaline phosphatase:** A normal WCC is typical in STB, indicating that the spinal infection is less likely to be pyogenic in nature, and an anaemic picture is common in the majority of STB presentations.^4,27,29,48^ An elevated alkaline phosphatase may also suggest the presence of STB. However, based on the small sample this result was reported in (21 cases), we cannot make a strong recommendation on its use.^29^ Finally, it must be noted that the Mantoux tuberculin skin test (TST) is not useful in TB endemic country or where the Bacillus Calmette–Guérin (BCG) vaccine is administered. HIV and other immunosuppressive conditions can lead to false negative readings of the TST.^26,49^ Therefore, the usefulness of the TST in people with suspected STB in South Africa is not supported by the literature.

#### Beneficial operational issues: Primary health care outreach and clinic support

**Terminology for people with suspicion of spinal tuberculosis:** As the patient is investigated further for STB, Jain suggests the label of a ‘spine under observation’ may be a useful term to use.^23^ We suggest that at the primary level of care this label would facilitate completion of investigations, decrease risk of inaccurate labelling of the patient and decrease the risk of assumption that the diagnosis has been confirmed until such time as the diagnosis is finalised.

**Role of doctor, physiotherapist and speech therapist:** Following the above investigations conducted by a clinical nurse at the primary level of care, further information could be gained with a referral for assessment to the outreach physiotherapist and doctor who visit the clinic, as well as the speech therapist if swallowing difficulties, stridor or hoarseness of voice are present in patients complaining of neck symptoms.^20,50,51^ Full neurological examination including sensation, reflexes and muscle power by the nurse, doctor, physiotherapist and/or speech therapist is essential, because early cases of STB can present with neurological signs of which the patient themselves are unaware.^12,52^ If the services of these professionals are not available at clinic level, referral should be made to the district hospital.

### Tools at district hospital level

#### Maximally utilising radiography

All patients with the suspicion of STB should be referred for radiographs (X-rays), which remain the mainstay of diagnosis because of their affordability and accessibility, particularly in developing countries,^24^ and despite the superior sensitivity and specificity of MRI.^4,5,22^ Polley and Dunn recommend that the entire spine be radiographed in all STB suspects.^53^ In addition, chest radiographs should be performed simultaneously, although it is possible that people with proven pulmonary tuberculosis may have unremarkable chest radiographs.^54^

Radiographs are useful for showing both vertebral destruction as well as soft tissue shadows. Jain noted that radiographs can reveal increased prevertebral soft tissue shadows in 7 out of 11 cases of cervical and cervicothoracic lesions.^55^ Rivas-Garcia et al. recommend that oblique views be included in order to detect early changes that involve anterolateral corners of the vertebra.^41^

The limitations of radiographs have been demonstrated in numerous studies with MRI shown to be more accurate for identifying the number of levels involved and extent of soft tissue involvement. In non-culture verified studies, 6% – 12% of lesions seen on MRI were not visible on radiographs.^5,17,56,57^ Jain noted in 49 cases of STB that, when investigated radiographically, the average number of involved vertebra reported was 2.6, compared to 3.2 on MRI investigations.^5,18,23,56,57^ However, in poor resource settings, MRI is often not available and therefore careful interpretation of radiograph findings is essential, especially as many of the differential conditions are difficult to differentiate from STB even on MRI.^24^

**Interpreting radiograph findings:** At the earliest stages of STB, radiographs may show no changes. With disease progression, lesions may be seen in the following patterns: paradiscal, anterior, central and posterior (Box 1^59^; Figure 1). The presence of calcification within an abscess is considered diagnostic of STB.^20^

Box 1 Types of spinal tuberculosis lesions.

**Paradiscal lesion (‘typical’):** Disc space narrowing, possibly with rarefaction of vertebral end plates and loss of cortical bone with or without paravertebral shadows from an abscess, progressing to vertebral collapse.^22,24,41,58,59^

**Anterior lesion:** Lesion is seen in one corner of the vertebra; it may be easier to see on oblique view.^41^ Its spread underneath the anterior longitudinal ligament can produce a scalloping of the anterolateral surface of the vertebral bodies, which may progress to vertebral body collapse. Soft tissue shadow from the abscess may be seen.^22,41,59^

**Central lesion:** This can often be confused with malignancy as it affects only the vertebral body, with the disc space unaffected. Progression leads to collapse of the whole vertebral body.^17,22,41^

**Posterior lesion:** The vertebral arch is affected, which is difficult to see on plain radiograph. There may be a soft tissue shadow from the abscess.^22,41,58,59^ Spinal tumour syndrome/intradural tuberculoma: There is no vertebral destruction and it is not visible with radiography alone.^5,53,60^

*Source*: Adapted from Ansari S, Amanullah M, Ahmad K, Rauniyar RK. Pott’s spine: Diagnostic imaging modalities and technology advancements. N Am J Med Sci. 2013;5(7):404–411. https://doi.org/10.4103/1947-2714.115775.^59^

**FIGURE 2:**
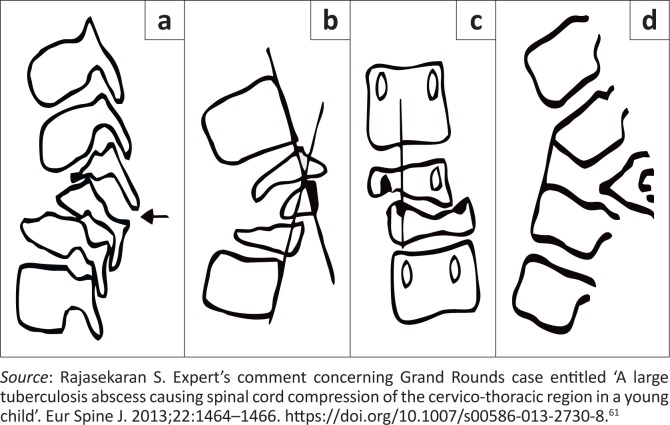
Diagram of the radiological signs for the ‘spine at risk’: (a) Separation of the facet joint: The facet joint dislocates at the level of the apex of the curve, causing instability and loss of alignment. In severe cases the separation can occur at two levels; (b) Posterior retropulsion: This is identified by drawing two lines along the posterior surface of the first upper and lower normal vertebrae. The diseased segments are found to be posterior to the intersection of the lines; (c) Lateral translation: This is confirmed when a vertical line drawn through the middle of the pedicle of the first lower normal vertebra does not touch the pedicle of the first upper normal vertebra; (d) Toppling sign: In the initial stages of collapse, a line drawn along the anterior surface of the first lower normal vertebra intersects the inferior surface of the first upper normal vertebra. ‘Tilt’ or ‘toppling’ occurs when the line intersects higher than the middle of the anterior surface of the first normal upper vertebra.

Importantly, radiographs can reveal spinal instability when they are evaluated using Rajasekaran’s ‘spine at risk’ signs (Figure 2).^61^

**Telemedicine support for interpretation of radiographic investigations:** Telemedicine support has been very effective in ophthalmology, with the Vula App assisting clinicians in remote areas to interpret test results and optimise diagnosis.^62^ Increased telemedicine support, in the form of assisting in reading radiographs of patients who have suspected STB, may enable earlier diagnosis of STB and increase the skills of primary care clinicians.

#### Ultrasound: A cheap, available and useful asset

District hospitals may have access to ultrasound, a cost-effective imaging modality. An abdominal ultrasound performed by an experienced clinician or ultrasonographer could assist in the exploration of extra-pulmonary TB (EPTB) sites or lymph nodes that can be found concomitantly with STB.^11,17,20,29^ STB, as a form of EPTB, can be accompanied by other forms of EPTB. If EPTB sites are identified and positively diagnosed using ultrasound, the likelihood of STB as a cause for back pain is increased. Further, given that treatment of EPTB is of the same duration as STB, the dual diagnosis ensures a patient with query STB receives adequate treatment. In the presence of accessible abscesses, ultrasound can guide a percutaneous biopsy, which can then be sent for culture and assessed with GeneXpert.^11,18,29^ Held et al., in their study comparing culture and GeneXpert in 71 biopsy samples, recently evaluated GeneXpert to have the following accuracies: sensitivity 95.6%, specificity 96.2%, positive predictive value 97.7% and negative predictive value 92.6%. The speed of reporting is a major advantage of GeneXpert.^6,63,64^ It should be noted that routine drainage of abscesses is not recommended, as this may result in sinus formation.^20,65,66^

#### Urine lipoarabinomannan for CD4 < 200

In patients living with HIV whose CD4 count is <200, the newly available urine Lipoarabinomannan (LAM) test is a useful adjunct to the diagnosis of TB with a positive predictive value of 88.5% and a negative predictive value of 87.1% for all TB when used alone.^43^ Therefore, it is not specific for STB but can be a useful adjunct to diagnosis, specifically in patients whose CD4 count is < 200.

### Tools at all levels of care: The vitally important element of treatment response as confirmation of diagnosis

Response to treatment is an important element to consider as part of the diagnostic process, because a positive response to treatment may be the only confirmation of diagnosis, as noted in many of the research studies that use this in practice. The gold standard of culture on CT-guided biopsy is also not 100% sensitive; thus, response to treatment is valuable in confirming accurate diagnosis and appropriate treatment. Consequently, it is essential that response to treatment be monitored in all people with a diagnosis of STB.

Positive response to treatment can be observed at four to six weeks^15,31,60^ with the following changes: normalising CRP and/or ESR, weight gain, decrease in symptoms (pain, neurological symptoms) and no further deterioration on radiograph (or MRI if possible).^17,18,32,55,58^ To accurately measure improvement, all of these measures need to have a baseline with which to compare. All cases of non-response must be investigated further without delay, with the possibility of drug-resistant STB or other differential diagnoses being made.^15,17,24,55^

### Referral from district to tertiary level of care

Clear referral criteria instil confidence in the referring clinician, as well as reducing unnecessary overload at the specialist level. Investigation at specialist level could include MRI, percutaneous CT-guided biopsy or surgical biopsy. The referral criteria in Box 2 are proposed in accordance with the literature.^6,20,26,50,61,66,67^

Box 2 Referral criteria for spinal tuberculosis.

**Referral for confirmation of suspected diagnosis:**

- In areas where MRI and CT-guided biopsy are accessible, all diagnoses of STB should be investigated with MRI and confirmed by biopsy.^20,22,25,26,32,53^ This is the current gold standard.
- In areas where CT-guided biopsy is not readily available, STB suspects who do not respond to treatment within six weeks should be referred for investigation.

**Referral for investigation:**

- Children with immature skeletons with suspected STB.
- Failure to respond to ATT within six weeks.
- Neurological deterioration or lack of improvement on ATT.
- Uncertainty of diagnosis.

**Urgent referral for surgery from all levels of care:**

- Spinal instability according to Rajasekaran’s ‘spine at risk’ signs.
- Kyphosis with Cobb angle >30°.
- Rapid deterioration of neurological signs, or paraplegia of rapid onset.
- Airway compromise or swallowing difficulties in cervical spine disease.
- Paraplegia with evidence on MRI of epidural pus and granulation tissue. compressing the spinal cord or signal cord change on MRI.
- Paravertebral abscess that increases in size despite ATT.
- Severe back or radicular pain.

MRI, magnetic resonance imaging; CT, computerised tomography; STB, spinal tuberculosis; ATT, anti-tuberculous treatment.

Referral for MRI investigation for those whose diagnosis is unclear is important because MRI detects STB in previously difficult-to-see locations like the craniocervical/occipitocervical and cervicothoracic junctions.^22^ MRI has been shown to have a sensitivity of 100% and a specificity of 88.2% with STB.^4^ MRI has also revealed that presentations, like central body lesions or skip lesions, are more common than previously thought. In a study utilising full spine MRI, Polley and Dunn showed a 16.3% incidence of skip (non-concomitant) lesions. It is notable that 15 of the 16 lesions identified were evident on plain radiograph as well as MRI scan in this non-blinded study.^53^

## Further recommendations

The data collected to inform understanding about STB is predominantly demographic and biomedical, with only one study found that used an Oswestry Disability Index.^68^ This reflects a large gap in the research in describing the lived experience of the path to diagnosis for people with this disease, as well as its social determinants. In addition, evidence from non-tertiary centres is lacking, and further research at a primary level will assist in understanding delays in diagnosis and addressing the key causes. It would be helpful for PHC and district hospital practice to have more research from which to develop protocols and guidelines.

The suggested diagnostic investigations are affordable and the costs insignificant when considered in comparison with the cost of surgical treatment for complicated STB and the cost of living with a disability. In Box 3, the 2017 prices of the suggested investigative PHC tests in South Africa are illustrated, demonstrating the low costs involved. Thus, it can be demonstrated that cost is not a valid barrier to implementing these tests and actions.

Box 3 Cost in 2017 of primary care tests.

**HIV, CD4 count and viral load (falls under standard HIV care):**

- R50 + R60 + R306 = R416

**Sputum (culture and GeneXpert):**

- R96 + R173 = R169

**ESR, CRP, platelets, WCC, Hb and alk phos:**

- R26 + R66 + R19 + R16 + R16 + R39 = R182

*Source*: Based on the National Health Laboratory Services District Hospitals request form (Cilliers S 2017, personal communication, Sept 25)

ESR, erythrocyte sedimentation rate; alk phos, alkaline phosphatase; CRP, C-reactive protein; WCC, white cell count.

Utilisation of these tests may facilitate the early diagnosis of STB. Access to a full multidisciplinary team at all district hospitals with support at remote clinics may also contribute to sustainability and quality of care for people with STB. Finally, outreach services at clinics reduce the cost and travel burden for all people but especially those with mobility difficulty and pain as in STB. In addition, given the complexity of the clinical reasoning process in diagnosing STB, there may be value in investing in experienced staff to improve retention rates.

There are some risks in increasing awareness of STB diagnosis. These include increased anxiety for those with back pain, unnecessary investigations, and the risk of false positive diagnoses and subsequently possible unnecessary drug side effects. These negative effects need to be weighed against the gains that could be attained through early diagnosis.^69,70^

## Conclusion

No single test can identify STB; however there are many clinical tools that assist the health care practitioner in the identification of early STB before complications develop. Although these tests are not specific for STB (i.e. negative results do not exclude STB and positive results do not confirm it), the diagnostic tools reviewed here provide the clinician with information to use in clinical reasoning around the suspicion of STB in an endemic country. The tests are available and affordable at PHC and district levels in South Africa, and their utilisation may optimise the early identification and timeous management of STB.
